# Spontaneous regression of B-cell acute lymphoblastic leukemia with PAX5 alterations at relapse: a case report

**DOI:** 10.1007/s00277-026-06825-4

**Published:** 2026-07-31

**Authors:** Vadim Lesan, Joerg Bittenbring, Sarah Altmeyer

**Affiliations:** Department of Hematology and Oncology, Saarland University Hospital, Kirrberger Street 100, 66421 Homburg, Germany

**Keywords:** Spontaneous regression, B-ALL, PAX5 alterations, Case report

## Abstract

Acute lymphoblastic leukemia is the most common pediatric hematological disease representing less than 1% of hematological diseases in adults. The prognosis of ALL improved significantly in the last decades. Almost all patients require a therapy at the time of diagnosis. Rare cases of spontaneous remission of ALL have been described. We report a case of B-ALL that underwent spontaneous remission after an episode of infection and describe the cytogenetic changes associated with this uncommon clinical presentation of B-ALL.

## Introduction

Acute lymphoblastic leukemia (ALL) is the most common pediatric hematological disease. The prognosis of the disease has changed dramatically over the last decades, with survival rates in the modern era exceeding 90% in children [1]. ALL exhibits a significant age-related prognostic disparity, with adult patients generally facing a less favorable outlook compared to children. Over the past few decades, advancements in treatment protocols and supportive care have led to substantial improvements in outcomes for both age groups [2].

B-cell ALL (B-ALL) is the most common ALL subtype, accounting for 75% of all cases. Generally, in both pediatric and adult populations, T-ALL is less frequent and is associated with a worse prognosis.

Almost universally, all patients require treatment at the time of diagnosis, as the uncontrolled proliferation of ALL blasts leads to anemia, thrombocytopenia with an increased risk of bleeding, and leukopenia with an increased risk of life-threatening infections. Spontaneous remission of ALL is a rare and not well-understood phenomenon that has garnered increasing interest in the medical literature [3, 4]. Case reports describing this phenomenon date back as early as 1878 and continue to appear periodically in the medical literature. Small case series on patients experiencing spontaneous remission of ALL have been recently published [5]. Most of these cases concern pediatric patients, although spontaneous remission has also been reported in adults [6, 7].

The pathophysiological processes leading to ALL remission remain unknown. Almost all case reports describe spontaneous remissions in patients who, at the time of diagnosis, suffered from infections (particularly bacteremia), received blood transfusions, or were treated with corticosteroids for other comorbidities [5]. The time from ALL diagnosis to spontaneous remission varies, ranging from a few days to several weeks and nearly all cases eventually relapse with a leukemia-free period following spontaneous remission between two weeks and 14 months [5].

Cases of ALL with spontaneous remission have been compared to prodromal ALL (or pre-ALL), in which the aleukemic phase is often associated with infections and can mimic spontaneous remission [3]. As such, pre-ALL is very similar to spontaneous remission ALL and should be carefully differentiated.

In this case report, we describe an adult patient with common B-ALL who experienced a transient complete remission of leukemia and report on cytogenetic changes associated with this uncommon clinical presentation of ALL.

### Initial presentation

A 47-year-old female patient consulted her general physician for a routine check-up four months after undergoing bariatric surgery for obesity. Due to complaints of fatigue, a complete blood count (CBC) was performed, revealing pancytopenia with hemoglobin (Hb) 9.9 g/dL, thrombocytopenia (58 Giga/L), and mild leukopenia (1.1 Giga/L). To exclude an underlying hematological malignancy, a bone marrow biopsy was performed by a hematologist, which revealed 90% bone marrow infiltration with B-acute lymphoblastic leukemia (B-ALL) with following immunohistochemical profile: CD34/CD20/CD10/CD19/terminal deoxynucleotidyl transferase (TdT) -positive and MPO/CD117/CD33/CD13-negative.

Three days after bone marrow biopsy, the patient developed fever and was urgently hospitalized in a secondary hospital for the administration of intravenous antibiotics. At presentation, fever was the solely symptom. Blood and urine cultures remained sterile. The febrile episode was treated with Piperacilin/Tazobactam for 7 days (after a total of 4 days of fever) and no corticosteroids were administered during the febrile episode.

### Diagnostic workup

Upon admission to our clinic, the broad-spectrum antibiotic therapy was continued. A peripheral blood smear performed on the day of admission revealed no blast cells. To confirm the diagnosis and conduct cytogenetic analysis, a new bone marrow biopsy was attempted. However, this resulted in a dry tap with histological evaluation showing no evidence of leukemia. Molecular studies on peripheral blood (PB) demonstrated clonality of immunoglobulin heavy chain (IgH) and T cell receptor (TCR) gamma/delta gene rearrangements. Flow cytometry analysis of PB identified 1% of CD45low, CD19/CD10/CD34/CD20-positive cells, which were negative for TdT and both surface and cytoplasmic immunoglobulin M (IgM).

Three days post-admission, a second bone marrow biopsy was performed. This showed no histological signs of B-cell acute lymphoblastic leukemia (B-ALL) and only 0.4% of CD45low, CD10/CD19/CD34-positive, TdT and sIgM-negative cells on flow cytometry analysis of the bone marrow.

**Fig. 1:**
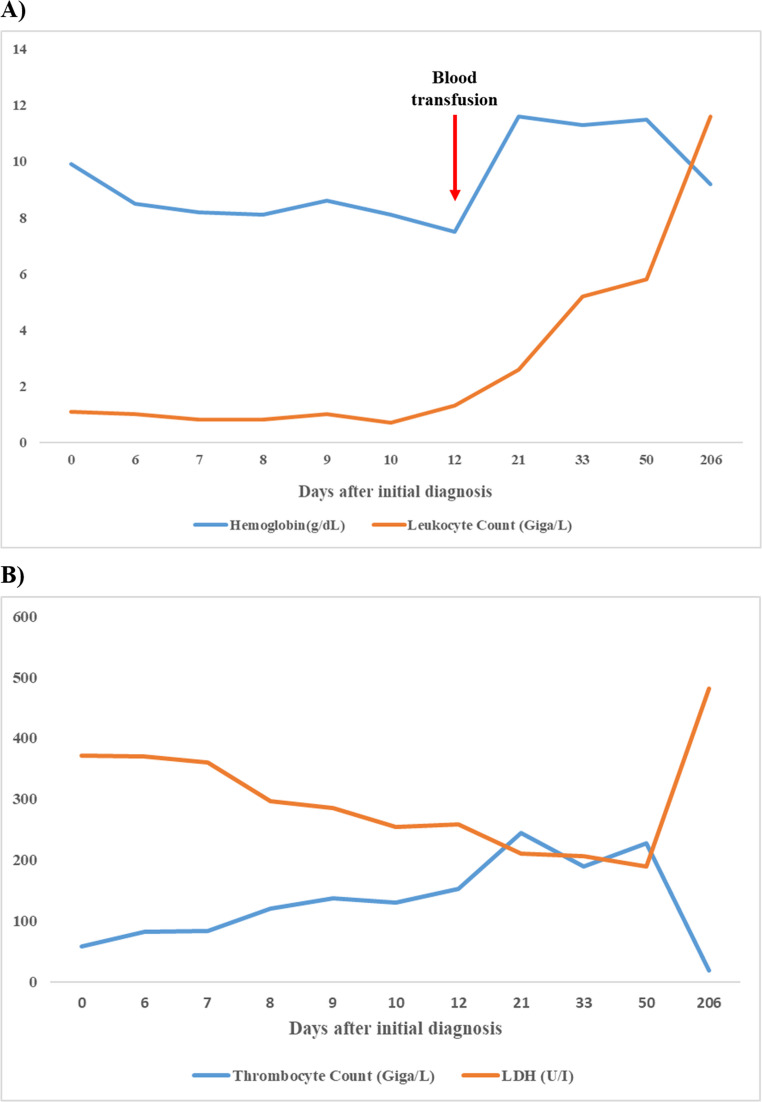
**A**) Changes in hemoglobin and leukocytes in peripheral blood before relapse. **B**) Changes in thrombocyte count and C-reactive protein levels in peripheral blood before relapse. Timeline presented as days after initial diagnosis of B-ALL

On the fourth day of hospitalization, a blood transfusion was administered to address progressive, symptomatic anemia. The thrombocyte count showed progressive recovery after initial diagnosis, with counts normalizing around day 12 after initial diagnosis (Fig. 1). Following resolution of the infection, an 18 F-fluorodeoxyglucose positron emission tomography-computed tomography (FDG PET-CT) scan was conducted, revealing no signs of lymphadenopathy or malignancy.

The patient was subsequently discharged and transitioned to follow-up care in an ambulatory setting.

### Pathology review and follow-up

The initial bone marrow biopsy, which had demonstrated the B-ALL, was reviewed in our pathology department by a hematopathologist specializing in leukemia, confirming the initial diagnosis of B-ALL.

One month later, a repeat bone marrow biopsy showed again no histological signs of leukemia. Bone marrow flow cytometry detected a small (0.4%) CD10/CD19/CD34/CD45low-positive and TdT/sIgM negative cell population, while molecular testing showed persistent clonal IgH rearrangement.

**Fig. 2:**
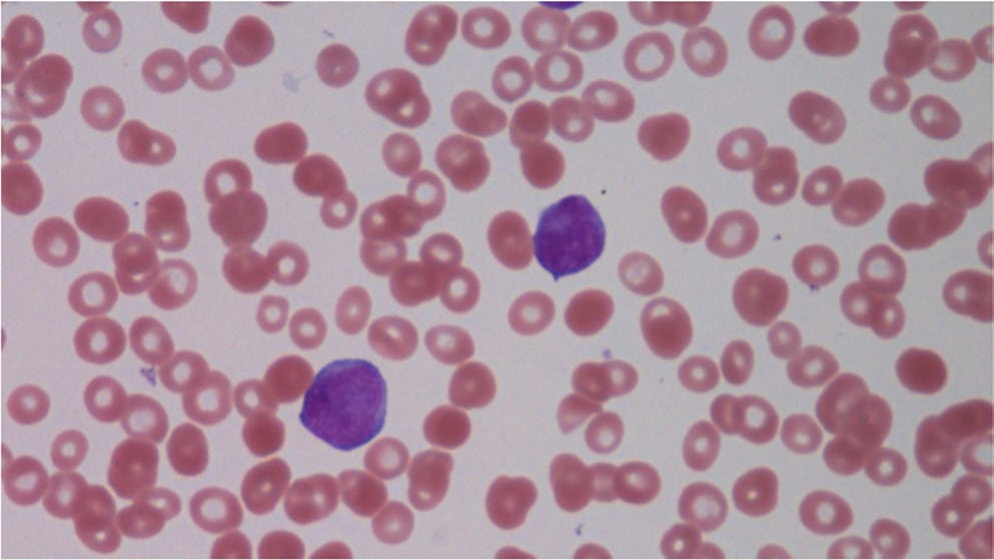
Microscopic image of a Giemsa-Wright-stained peripheral blood smear performed on day 206 after diagnosis (at relapse)

**Fig. 3:**
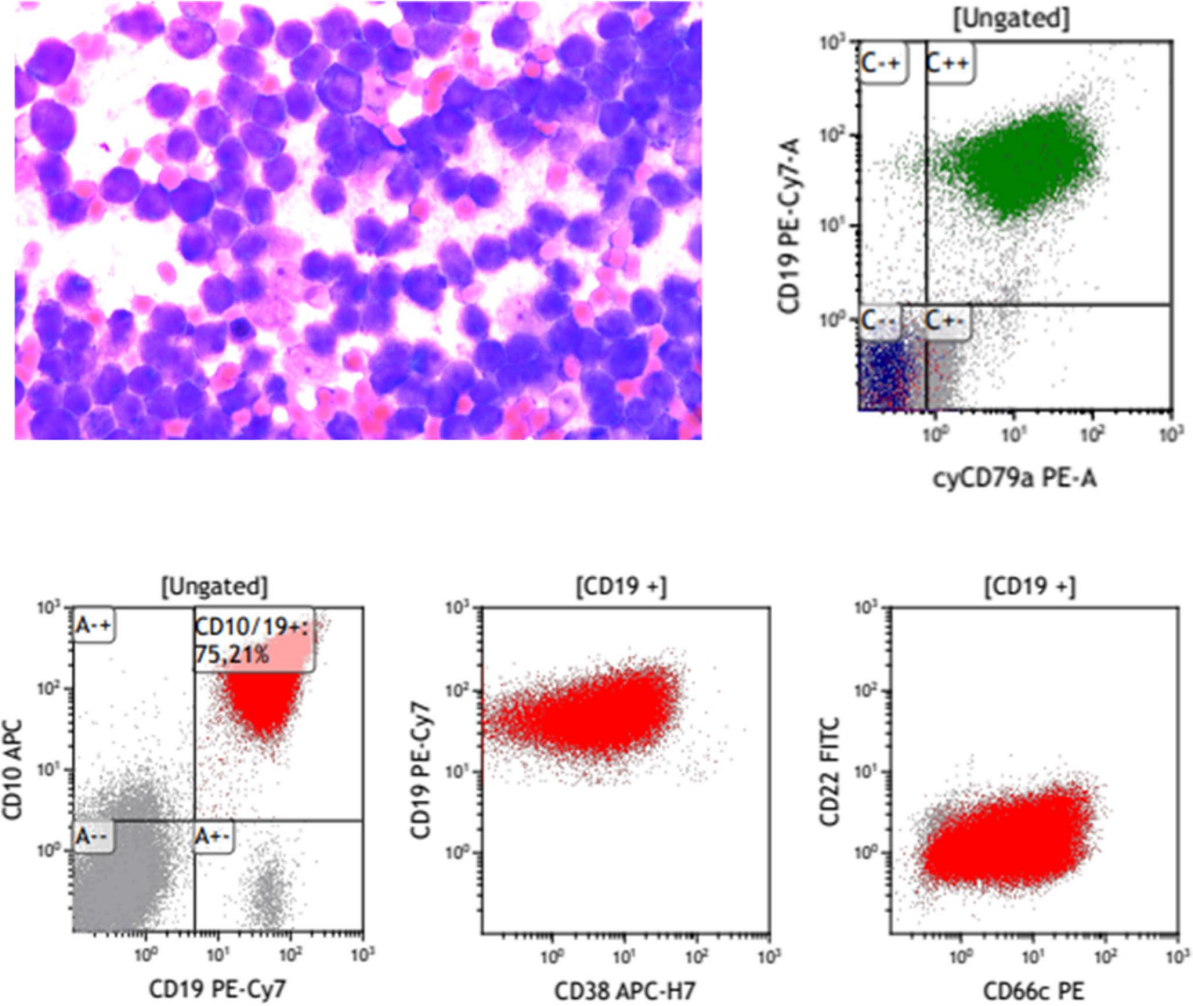
Cytology and flow cytometry on bone marrow aspiration on day 206 after initial diagnosis (at relapse)

Following last ambulatory presentation, the patient was lost to follow-up but re-presented six months later to our emergency department with severe anemia (Hb 8.1 g/dL) and thrombocytopenia (14 Giga/L). The leukocyte count was normal (5.1 Giga/L). Peripheral blood smear examination revealed 35% blasts, raising suspicion of a B-ALL relapse (Fig. 2). The bone marrow biopsy confirmed a 90% infiltration with leukemic blasts. Bone marrow flow cytometry analysis demonstrated a CD45low/CD10/CD19/CD20/CD34/TdT-positive phenotype, while histology confirmed relapsed common B-ALL (Fig. 3). A different clonal IgH rearrangement was observed at this timepoint.

### Cytogenetic and molecular analysis

At the time of relapse, the chromosomal analysis revealed a complex karyotype. Fluorescence in situ hybridization did not detect a BCR-ABL1 rearrangement, and reverse transcription polymerase chain reaction (RT-PCR) was negative for BCR-ABL1 fusion products. Additional FISH analysis identified a CDKN2A deletion, MYC amplification, 5p15 and 5q31 amplification, and a 13q14 deletion. Using the Gene Expression Classification Algorithm (ALLCatchR), the leukemia was classified as common B-ALL with PAX5 alterations [8].

### Therapy and follow-up

Since B-ALL with PAX5 alterations has an unfavorable prognosis in adult patients, we commenced a therapy as per GMALL recommendations and initiated a bone marrow transplant donor search [9]. The bone marrow biopsy after first induction therapy revealed a complete cytological remission. The therapy will continue as per GMALL recommendations, and a marrow transplantation will be commenced as soon as a donor will be available.

## Discussion

Spontaneous remission (SR) of acute lymphoblastic leukemia (ALL) is an exceptionally rare phenomenon, though well-documented case reports exist in the medical literature [5]. The underlying pathophysiological mechanisms remain largely elusive, as most reported cases describe an association with severe infections, blood transfusions (through graft-versus-leukemia effect), and the administration of corticosteroids [5]. Although, spontaneous remission induced by the administration of corticosteroids is not considered true SR (given their well-established anti-leukemic effects), endogenic corticosteroids released during a stressful situation like infection could induce such changes [10].

Our case of SR in B-ALL supports the association with severe infection and less probable with blood transfusion (administered 4 days after remission observation), while also providing additional data on the time to relapse and the cytogenetic landscape of relapsed ALL following SR.

A noteworthy consideration is the possibility that our case represents a prodromal form of ALL (pre-ALL), a pancytopenic phase preceding the overt diagnosis of leukemia. Several clinical features of pre-ALL resemble our case, including female predominance and presentation with fever and fatigue [3]. However, while pre-ALL is typically characterized by a hypoplastic bone marrow, our case displayed normal hematopoietic cellularity, distinguishing it from classical pre-ALL presentations. Pre-ALL is rarely recognized in adults, and many such cases may be misclassified as aplastic anemia or hypoplastic myelodysplastic neoplasm (MDS) and subsequently treated as such [11]. The distinction between SR and pre-ALL might be very difficult, especially with the possibility that both could appear independent and precede each other representing a biological continuum of the same disease. As such, the distinction of SR and pre-ALL might represent simply a timing sampling problem and not true biological differences [12, 13]. Flow cytometry is also limited in this context, as it lacks both sensitivity and specificity for distinguishing residual leukemic blasts from regenerating hematogones. In our case, TdT expression was positive at diagnosis and relapse, but negative during the intermediate phase, which is more consistent with transient hematogone regeneration than with persistent leukemic disease. Although the presence of a clonal IgH rearrangement during cytological remission raises the possibility of a pre-leukemic clone, this finding may also occur as a reactive monoclonal B-cell proliferation following infection. Importantly, the IgH rearrangement at relapse differed from that detected during the remission period, supporting an infection-associated reactive clone rather than a true pre-ALL state. Interestingly, our case also demonstrated clonality of T-cell receptor (TCR) gamma/delta (γδ) rearrangements, raising the possibility that γδ T cells may have played a role in leukemia control. This γδ T-cell expansion could have been triggered either as a response to infection, as a reaction to leukemic cells or as a consequence of IgH/TCR cross-lineage rearrangement without any pathological significance [14]. It is well documented that γδ T cells possess unique anti-leukemic properties, including (a) MHC-independent recognition and cytotoxicity against leukemic blasts, making them effective even when CD8 + T cell-mediated immunity is evaded, (b) production of pro-inflammatory cytokines, such as IFN-γ and TNF-α, which enhance anti-leukemic immunity by activating NK cells, dendritic cells, and other immune components and (c) IL-17 secretion, which has been reported to exert anti-leukemic effects [15]. However, whether γδ T cells contributed to spontaneous remission in our case remains unknown and warrants further investigation.

At relapse, our case provides additional data on the cytogenetic and molecular subtypes of B-ALL capable of undergoing spontaneous remission. The PAX5-altered B-ALL identified in our patient represents a provisional entity in the International Consensus Classification (ICC; Arber et al., Blood 2022) and falls under the category of “B-ALL with other defined genetic alterations” in the WHO HAEM5 classification [16, 17].

B-ALL with PAX5 alterations accounts for approximately 7.5% of all B-ALL cases and occurs at the following frequencies: 3–9% in patients ≤ 15 years; 9% in patients aged 16–39 years and 3% in patients ≥ 40 years [16]. The prognosis of PAX5-altered B-ALL is intermediate in pediatric patients but poor in adults. Most PAX5 altered leukemias show a range of genetic abnormalities, including rearrangements, point mutations (excluding P80R), intragenic amplifications [17].

One important limitation of our case is the lack bone marrow images and cytogenetic analysis at the time of the initial diagnosis. As such, we cannot assess the causative role of PAX5 alterations with spontaneous remission in ALL. Despite this limitation, similar cytogenetic alterations have been previously described in pediatric patients with ALL experiencing a preleukemic aplastic phase [18].

## Conclusion

This case contributes to the limited but growing body of evidence on spontaneous remission of ALL, providing new insights into its possible pathophysiology, pre-leukemic states (pre-ALL), and the molecular characteristics of relapsed disease. Future studies should focus on identifying immune mechanisms—such as γδ T-cell responses—that could potentially be harnessed for therapeutic benefit in ALL.

## Data Availability

No datasets were generated or analysed during the current study.

## References

[1] Sasaki K, Jabbour E, Short NJ, Jain N, Ravandi F, Pui CH et al (2021) Acute lymphoblastic leukemia: a population-based study of outcome in the United States based on the surveillance, epidemiology, and end results (SEER) database, 1980–2017. Am J Hematol [Internet]. [cited 2025 Feb 2];96(6):650. Available from: https://pmc.ncbi.nlm.nih.gov/articles/PMC9517941/10.1002/ajh.26156 PMC9517941 33709456

[2] Yi M, Zhou L, Li A, Luo S, Wu K (2020) Global burden and trend of acute lymphoblastic leukemia from 1990 to 2017. Aging (Albany NY) [Internet]. [cited 2025 Feb 2];12(22):22869. Available from: https://pmc.ncbi.nlm.nih.gov/articles/PMC7746341/10.18632/aging.103982 PMC7746341 33203796

[3] Höres T, Wendelin K, Schaefer-Eckart K (2018) Spontaneous remission of acute lymphoblastic leukemia: a case report. Oncol Lett [Internet]. [cited 2025 Feb 2];15(1):115–20. Available from: http://www.spandidos-publications.com/10.3892/ol.2017.7288/abstract 10.3892/ol.2017.7288 PMC5738709 29285190

[4] Cherif A, Saada V, Bouatay A (2022) Case report spontaneous remission of early T-cell precursor acute lymphoblastic leukemia. F1000Research [Internet]. [cited 2025 Feb 2];11:1407. Available from: https://f1000research.com/articles/11-1407

[5] Kirtek T, Hamdan H, Van Arnam JS, Park S, Kovach AE, Pillai V et al (2023) Spontaneous remission of acute lymphoblastic leukemia: a series of nine cases and a review of literature. Int J Lab Hematol [Internet]. [cited 2025 Feb 2];45(4):489–95. Available from: https://pubmed.ncbi.nlm.nih.gov/36806637/10.1111/ijlh.14042 36806637

[6] Gilbert A, Tan J, Nadimpalli S, Orkusyan R, Fernandez ZI, Oak J et al (2023) B-lymphoblastic leukemia with transient spontaneous remission in the setting of severe group A streptococcus infection. J Hematop [Internet]. [cited 2025 Feb 2];16(4):223–6. Available from: https://link.springer.com/10.1007/s12308-023-00564-5 10.1007/s12308-023-00564-5 38175433

[7] McCormick BJ, Imran H (2024) Spontaneous remission of acute lymphoblastic leukemia following Candida tropicalis Fungemia. Cureus [Internet]. [cited 2025 Feb 2];16(6):e62435. Available from: https://pmc.ncbi.nlm.nih.gov/articles/PMC11249080/10.7759/cureus.62435 PMC11249080 39011219

[8] Beder T, Hansen BT, Hartmann AM, Zimmermann J, Amelunxen E, Wolgast N et al (2023) The gene expression classifier ALLCatchR identifies B-cell precursor ALL subtypes and underlying developmental trajectories across age. Hemasphere [Internet]. [cited 2025 Feb 2];7(9):E939. Available from: https://pubmed.ncbi.nlm.nih.gov/37645423/10.1097/HS9.0000000000000939 PMC10461941 37645423

[9] Gokbuget N, Hoelzer D, Arnold R, Böhme A, Bartram CR, Freund M et al (2000) Treatment of Adult ALL according to protocols of the German Multicenter Study Group for Adult ALL (GMALL). Hematol Oncol Clin North Am [Internet]. [cited 2025 Feb 2];14(6):1307–25. Available from: https://pubmed.ncbi.nlm.nih.gov/11147225/10.1016/s0889-8588(05)70188-x 11147225

[10] Inaba H, Pui CH (2010) Glucocorticoid use in acute lymphoblastic leukaemia. Lancet Oncol [Internet]. [cited 2025 Nov 20];11(11):1096–106. Available from: https://pubmed.ncbi.nlm.nih.gov/20947430/10.1016/S1470-2045(10)70114-5 PMC3309707 20947430

[11] Sohn SK, Suh JS, Lee J, Lee KB (1998) Pancytopenic prodrome (pre-ALL) of acute lymphoblastic leukemia in adults: possible pathogenesis. Korean J Intern Med [Internet]. [cited 2025 Feb 2];13(1):64. Available from: https://pmc.ncbi.nlm.nih.gov/articles/PMC4531931/10.3904/kjim.1998.13.1.64 PMC4531931 9538635

[12] Höres T, Wendelin K, Schaefer-Eckart K (2018) Spontaneous remission of acute lymphoblastic leukemia: a case report. Oncol Lett [Internet]. [cited 2025 Nov 20];15(1):115–20. Available from: https://pubmed.ncbi.nlm.nih.gov/29285190/10.3892/ol.2017.7288 PMC5738709 29285190

[13] Boonchalermvichian C, Xie Y, Brynes RK, Siddiqi IN (2012) Spontaneous, transient regression of B lymphoblastic leukemia in an adult patient: a variant presentation of prodromal/pre-ALL. Leuk Res [Internet]. [cited 2025 Nov 20];36(3). Available from: https://pubmed.ncbi.nlm.nih.gov/22129477/10.1016/j.leukres.2011.10.030 22129477

[14] Kelm M, Darzentas F, Darzentas N, Kotrova M, Wessels W, Bendig S et al (2023) Dominant T-cell receptor delta rearrangements in B-cell precursor acute lymphoblastic leukemia: leukemic markers or physiological γδ T repertoire? Hemasphere [Internet]. Sep 1 [cited 2025 Nov 20];7(9):E948. Available from: https://pubmed.ncbi.nlm.nih.gov/37670805/10.1097/HS9.0000000000000948 PMC10476800 37670805

[15] Hu Y, Hu Q, Li Y, Lu L, Xiang Z, Yin Z et al (2023) γδ T cells: origin and fate, subsets, diseases and immunotherapy. Signal Transduction and Targeted Therapy [Internet]. [cited 2025 Feb 2];8(1):1–38. Available from: https://www.nature.com/articles/s41392-023-01653-8 10.1038/s41392-023-01653-8 PMC10663641 37989744

[16] Arber DA, Orazi A, Hasserjian RP, Borowitz MJ, Calvo KR, Kvasnicka HM et al (2022) International consensus classification of myeloid neoplasms and acute leukemias: integrating morphologic, clinical, and genomic data. Blood 140(11):1200–28. 10.1182/blood.2022015850 35767897 10.1182/blood.2022015850 PMC9479031

[17] Khoury JD, Solary E, Abla O, Akkari Y, Alaggio R, Apperley JF et al (2022) The 5th edition of the World Health Organization Classification of Haematolymphoid Tumours: Myeloid and Histiocytic/Dendritic Neoplasms. Leukemia [Internet]. [cited 2025 Feb 2];36(7):1703–19. Available from: https://www.nature.com/articles/s41375-022-01613-1 10.1038/s41375-022-01613-1 PMC9252913 35732831

[18] Horsley SW, Colman S, McKinley M, Bateman CM, Jenney M, Chaplin T et al (2008) Genetic lesions in a preleukemic aplasia phase in a child with acute lymphoblastic leukemia. Genes Chromosomes Cancer [Internet]. [cited 2025 Nov 20];47(4):333–40. Available from: https://pubmed.ncbi.nlm.nih.gov/18181181/10.1002/gcc.20537 18181181

